# GLP-1 RA Use and Major Adverse Cardiovascular Events in Patients With Monoclonal Gammopathy of Undetermined Significance

**DOI:** 10.1001/jamanetworkopen.2025.17541

**Published:** 2025-06-30

**Authors:** Kuan-Yu Chi, Junmin Song, Shweta Desphande, Pei-Lun Lee, Anushri Soni, Antony Gonzales-Uribe, Yasmin Lessa, Ahmed Ashraf Morgan, Yu Chang, Yu-Shiuan Lin, Zafer Akman, Armin Nouri, Raiza Rossi, Golsa Babapour, Dimitrios Varrias, Terri Parker, Lauren A. Baldassarre, Alokkumar Jha, Eli Muchtar, Sarah C. Hull, Jennifer M. Kwan, Michael G. Nanna

**Affiliations:** 1 Department of Medicine, Jacobi Medical Center, Albert Einstein College of Medicine, Bronx, New York; 2 Section of Neurosurgery, Department of Surgery, National Cheng Kung University Hospital, College of Medicine, National Cheng Kung University, Tainan, Taiwan; 3 Department of Education, Center for Evidence-Based Medicine, Taipei Medical University Hospital, Taipei, Taiwan; 4 Section of Cardiovascular Medicine, Yale School of Medicine, New Haven, Connecticut; 5 Department of Internal Medicine, Section of Hematology, Yale University School of Medicine and Yale Cancer Center, New Haven, Connecticut; 6 Weill Cornell Medicine, New York, New York; 7 Division of Hematology, Mayo Clinic, Rochester, Minnesota

## Abstract

**Importance:**

Monoclonal gammopathy of undetermined significance (MGUS) is associated with an increased risk of cardiovascular disease. Glucagon-like peptide-1 (GLP-1) receptor agonists (RAs) have demonstrated cardiorenal benefits in patients with type 2 diabetes, but their effectiveness in patients with MGUS remains unexplored.

**Objective:**

To assess the effectiveness of GLP-1 RAs for primary prevention of major adverse cardiovascular and cerebrovascular events (MACCE) in patients with MGUS and diabetes.

**Design, Setting, and Participants:**

This retrospective cohort study used a propensity score–matched analysis of data from the TriNetX Global Database, encompassing patients diagnosed with diabetes and MGUS between January 1, 2018, and January 13, 2023. Patients with prior heart failure (HF), ischemic heart disease, coronary revascularization, or stroke or transient ischemic attack before MGUS diagnosis were excluded. The cohort was divided into 2 groups: GLP-1 RA users and nonusers at baseline. After 1:1 propensity score matching, GLP-1 RA users and nonusers were compared up to 5 years from the MGUS diagnosis date. Data analyses were completed January 19, 2025.

**Exposure:**

GLP-1 RA use within 1 year before MGUS diagnosis.

**Main Outcomes and Measures:**

The primary end point was MACCE, defined as a composite of all-cause mortality, new-onset HF, acute coronary syndrome, and stroke or transient ischemic attack. Secondary end points included individual MACCE components, decompensated HF, and acute kidney injury or end-stage kidney disease.

**Results:**

A total of 4871 patients with MGUS (mean [SD] age, 68.9 [10.1] years; 2366 [48.5%] male) were included (473 GLP-1 RA users and 4398 non-users). A total of 460 users were matched to 460 nonusers, with balanced characteristics (mean [SD] age, 65.0 [10.6] vs 65.1 [11.0] years; 229 [49.7%] male vs 234 [50.8%] male), including 14 patients (3.0%) vs 13 patients (2.8%) identifying as Asian, 8 (21.3%) vs 92 (20.0%) as Black or African American, 25 patients (5.4%) vs 20 patients (4.3%) as Hispanic or Latino, and 243 patients (52.8%) vs 250 patients (54.3%) as White. GLP-1 RA use was associated with a significantly lower risk of MACCE (hazard ratio [HR], 0.75; 95% CI, 0.60-0.93). Significant reductions were also observed in all-cause mortality (HR, 0.57; 95% CI, 0.37-0.87), new-onset HF (HR, 0.69; 95% CI, 0.54-0.90), decompensated HF (HR, 0.60; 95% CI, 0.43-0.84), and acute kidney injury or end-stage kidney disease (HR, 0.73; 95% CI, 0.57-0.92).

**Conclusions and Relevance:**

The findings of this cohort study of GLP-1 RA use vs no use in patients with MGUS and diabetes suggest the potential of GLP-1 RA for primary prevention of MACCE. These findings warrant further investigation in prospective randomized trials.

## Key Points

**Question:**

Is glucagon-like peptide-1 (GLP-1) receptor agonist (RA) use associated with a major adverse cardiovascular and cerebrovascular events (MACCE) in patients with monoclonal gammopathy of undetermined significance and type 2 diabetes without prior cardiovascular disease?

**Findings:**

In this cohort study including 4871 patients, GLP-1 RA use was associated with significant reductions in MACCE, all-cause mortality, new-onset heart failure, decompensated heart failure, and acute kidney injury or end-stage kidney disease over a median follow-up of 3.2 years.

**Meaning:**

These findings suggest that GLP-1 RA use may provide primary prevention benefits for reducing MACCE in patients with monoclonal gammopathy of undetermined significance and diabetes.

## Teaser

This cohort study uses propensity score matching to assess the effectiveness of glucagon-like peptide-1 (GLP-1) receptor agonists (RAs) for primary prevention of major adverse cardiovascular and cerebrovascular events in patients with monoclonal gammopathy of undetermined significance and diabetes.

## Introduction

Monoclonal gammopathy of undetermined significance (MGUS) is a premalignant condition characterized by the presence of clonal plasma cells without evidence of multiple myeloma (MM) or other malignant lymphoproliferative diseases.^1^ The prevalence of MGUS increases gradually with age, affecting approximately 3.2% of individuals aged 50 years or older, and rising to 7.5% in those aged 85 years and older.^2^

While MGUS is typically asymptomatic and indolent, it is associated with an increased risk of several comorbidities beyond its progression to lymphoproliferative disorders.^3,4,5^ Notably, individuals with MGUS have an elevated risk of cardiovascular (CV) morbidity and mortality, compared with individuals without MGUS.^6,7^ The underlying mechanism for these observations may involve the chronic inflammatory state of MGUS,^8^ characterized by the upregulation of serum biomarkers (such as osteoprotegerin and receptor activator of nuclear factor-κB)^9^ and cytokines (such as interleukin-6),^10^ as well as potential direct infiltration of paraproteins into myocardial and renal tissues.^11^ A 2022 Danish population–based study highlighted the association of MGUS with a spectrum of CV diseases, including heart failure (HF), atrial fibrillation, cor pulmonale, myocardial infarction, venous thromboembolism, and valvular heart disease (VHD).^12^ These findings underscore the need for optimal management of CV risk factors to mitigate future CV events in this population.

Glucagon-like peptide-1 (GLP-1) receptor agonists (RAs) have demonstrated remarkable effectiveness in reducing CV events through their well-established cardiorenal and metabolic benefits.^13,14,15,16^ Emerging evidence also suggests potential survival benefits of GLP-1 RAs in cancer populations.^17,18,19,20^ Furthermore, data from the US Department of Veterans Affairs database showed that GLP-1 RA use in patients with MGUS and type 2 diabetes was associated with a significant 65% reduction in progression to MM, implying a possible chemopreventive role for GLP-1 RA.^21^ Despite these promising findings, the impact of GLP-1 RAs on CV outcomes in patients with MGUS remains unexplored. Hence, the aim of our study is to investigate the effectiveness associated with GLP-1 RAs in reducing future major adverse cardiac and cerebrovascular events (MACCE) in MGUS patients with diabetes.

## Methods

### Data Source

We conducted a retrospective cohort study using propensity score (PS) matching based on aggregate data from the TriNetX Global Collaborative Network, a federated database that aggregates real-time electronic health record (EHR) data from 142 health care organizations, comprising more than 155 million individuals.^22^ The TriNetX database operates under the Health Insurance Portability and Accountability Act (HIPAA) Privacy Rule as a limited dataset, which means that all patient data in the network is deidentified in adherence with HIPAA standards,^22^ excluding direct patient identifiers, such as names, social security numbers, and contact information, ensuring the protection of patient privacy. Consequently, research conducted using TriNetX data does not require institutional review board approval and informed consent, in accordance with the institutional review board of National Cheng Kung University Hospital. This study was conducted in accordance with the Strengthening the Reporting of Observational Studies in Epidemiology (STROBE) reporting guideline. Most participating health care organizations are based in the US.^22^ TriNetX uses a standardized data mapping process to convert health care data from various sources (EHR, claims data, and external death registries), into uniform terminologies, such as *International Statistical Classification of Diseases, Tenth Revision, Clinical Modification (ICD-10-CM)*, RxNorm, Anatomical Therapeutic Chemical code, and Current Procedure Terminology (CPT). Sex, race, and ethnicity were classified based on documentation in EHR, typically derived from patient self-report at the time of clinical encounter. Missing data in sex were categorized as unknown sex, and missing race or ethnicity were classified as unknown race or ethnicity. Researchers build up cohort queries on the TriNetX online portal using these standardized terminologies. Once a cohort is designed, the query is processed by TriNetX’s Advanced Analytics Platform, which provides built-in statistical tools for analysis. Numerous studies have successfully conducted comparative effectiveness research using TriNetX,^23,24,25,26,27^ including in the context of CV disease research.^28,29,30,31^

### Study Population and Design

We identified adult patients aged 18 years or older with type 2 diabetes and a new diagnosis of MGUS between January 1, 2018, and January 13, 2023. Patients with a prior or current diagnosis of MM (serum M protein ≥3 g/dL or bone marrow clonal plasma cells ≥10%), a history of polyclonal gammopathy, lymphoproliferative disorders, or amyloidosis were excluded. Additionally, we excluded patients with a history of HF, ischemic heart disease, coronary revascularization, or stroke or transient ischemic attack (TIA) prior to their incident MGUS diagnosis to satisfy the primary prevention design. We also excluded patients with contraindications for GLP-1 RA use prior to MGUS diagnosis, including acute or chronic pancreatitis, thyroid cancer, gastroparesis, and end stage kidney disease (ESKD). Patients were subsequently subdivided into 2 groups: GLP-1 RA users and nonusers. GLP-1 RA users were defined as individuals with a diagnosis of type 2 diabetes and having at least 1 prescription for a GLP-1 RA within 1 year before the MGUS diagnosis. GLP-1 RA nonusers were those who were treated with any non–GLP-1 RA antidiabetic medications, including metformin, dipeptidyl peptidase-4 inhibitors (DPP4Is), insulin, sulfonylureas, sodium-glucose cotransporter 2 inhibitors (SGLT2Is), thiazolidinediones, or alpha-glucosidase inhibitors within 1 year of the MGUS diagnosis. Those who had any record of GLP-1 RA use at any time before the MGUS diagnosis were excluded from the GLP-1 RA nonuser group. The details for cohort query are presented in eMethods 1 in Supplement 1.

### Statistical Analysis

Patients’ baseline covariates, including patient demographics, comorbidities, medications, procedures, health care encounters, and laboratory data, were gathered from the period between 1 day to 1 year prior to the incident diagnosis of MGUS. To address differences in baseline characteristics between groups, a PS-matched analysis was conducted to match the 2 cohorts in a 1:1 ratio using the greedy nearest-neighbor algorithm with a caliper of 0.1 pooled SDs. Differences in baseline characteristics between the 2 groups were compared using standardized mean differences (SMDs) after PS matching, with an SMD less than 0.10 indicating balanced characteristics. Descriptive statistics between GLP-1 RA users and nonusers before and after PS matching were reported for both continuous and dichotomous variables. We used independent-sample *t* tests for comparisons before PS matching, presented as means and SDs, and χ^2^ tests for to compare the cohorts after PS matching, reported as numbers and percentages. Inferential statistics of independent *t* tests and SMD were presented in the pre– and post–PS-matching cohorts, respectively. Covariates that were incorporated in the PS matching are presented in Table 1. When there were multiple values, TriNetX extracted the closest one to index date for matching.

**Table 1.** Baseline Characteristics of Patients With Monoclonal Gammopathy of Undetermined Significance Before and After PS Matching

Characteristic	Before PS Matching			After PS Matching^a^		
	Patients by GLP-1 RA Use, No. (%)		*P* value	Patients by GLP-1 RA Use, No. (%)		SMD
	Yes (n = 473)	No (n = 4398)		Yes (n = 460)	No (n = 460)	
Age at index, mean (SD), y	64.7 (10.7)	69.0 (10.8)	<.001	65.0 (10.6)	65.1 (11.0)	0.003
Race and ethnicity						
Asian	14 (3.0)	199 (4.5)	.11	14 (3.0)	13 (2.8)	0.013
Black or African American	103 (21.8)	973 (22.1)	.86	98 (21.3)	92 (20.0)	0.032
Hispanic or Latino	26 (5.5)	201 (4.6)	.36	25 (5.4)	20 (4.3)	0.050
White	250 (52.9)	2205 (50.1)	.26	243 (52.8)	250 (54.3)	0.031
Sex						
Male	232 (49.0)	2134 (48.5)	.52	229 (49.7)	234 (50.8)	0.036
Female	230 (48.6)	2064 (46.9)	.48	221 (48.0)	210 (45.7)	0.048
Diagnosis						
Essential hypertension	319 (67.4)	3025 (68.8)	.55	308 (67.0)	306 (66.5)	0.009
Chronic kidney disease	152 (32.1)	1623 (36.9)	.04	149 (32.4)	148 (32.2)	0.005
Atrial fibrillation and flutter	24 (5.1)	326 (7.4)	.06	24 (5.2)	28 (6.1)	0.038
Morbid obesity	87 (18.4)	378 (8.6)	<.001	79 (17.2)	91 (19.8)	0.067
Chronic lower respiratory diseases	84 (17.8)	679 (15.4)	.19	78 (17.0)	79 (17.2)	0.006
Malignant neoplasms						
Digestive organs	<10 (2.1)	80 (1.8)	.65	<10 (2.2)	<10 (2.2)	<0.001
Melanoma and other skin	17 (3.6)	109 (2.5)	.15	17 (3.7)	16 (3.5)	0.012
Breast	10 (2.1)	113 (2.6)	.55	10 (2.2)	12 (2.6)	0.028
Urinary tract	10 (2.1)	74 (1.7)	.49	<10 (2.2)	<10 (2.2)	<0.001
Bronchus and lung	10 (2.1)	24 (0.5)	<.001	<10 (2.2)	<10 (2.2)	<0.001
Other venous embolism and thrombosis	13 (2.7)	138 (3.1)	.64	13 (2.8)	11 (2.4)	0.027
Hyperlipidemia	196 (41.4)	1753 (39.9)	.51	187 (40.7)	197 (42.8)	0.044
Medication						
β-blocking agents	187 (39.5)	1715 (39.0)	.82	183 (39.8)	188 (40.9)	0.022
ACE inhibitors	145 (30.7)	1466 (33.3)	.24	143 (31.1)	134 (29.1)	0.043
Angiotensin II inhibitors	141 (29.8)	1251 (28.4)	.53	135 (29.3)	127 (27.6)	0.039
Calcium channel blockers	160 (33.8)	1701 (38.7)	.04	157 (34.1)	165 (35.9)	0.036
Statins	299 (63.2)	2681 (61.0)	.34	291 (63.3)	285 (62.0)	0.027
Potassium sparing or combinations diuretics	37 (7.8)	323 (7.3)	.71	37 (8.0)	39 (8.5)	0.016
SGLT2 inhibitors	90 (19.0)	286 (6.5)	<.001	79 (17.2)	75 (16.3)	0.023
Metformin	232 (49.0)	2487 (56.5)	.002	225 (48.9)	196 (42.6)	0.127
Insulin	245 (51.8)	1857 (42.2)	<.001	235 (51.1)	228 (49.6)	0.030
DPP4 inhibitors	35 (7.4)	673 (15.3)	<.001	35 (7.6)	35 (7.6)	<0.001
Sulfonylureas	109 (23.0)	1055 (24.0)	.65	105 (22.8)	101 (22.0)	0.021
Thiazolidinediones	31 (6.6)	183 (4.2)	.02	29 (6.3)	26 (5.7)	0.028
Anticoagulants	103 (21.8)	1103 (25.1)	.11	100 (21.7)	110 (23.9)	0.052
Aspirin	124 (26.2)	1023 (23.3)	.15	120 (26.1)	121 (26.3)	0.005
Laboratory values						
Hemoglobin A_1c_, %						
Mean (SD), %	7.5 (1.7)	7.1 (1.5)	<.001	7.5 (1.7)	7.4 (1.7)	0.057
≤8.5%	95 (20.1)	532 (12.1)	<.001	90 (19.6)	79 (17.2)	0.062
Glomerular filtration rate, mL/min/1.73 m^2^						
Mean (SD)	60.5 (29.4)	58.4 (27.7)	.19	60.5 (29.0)	57.8 (27.2)	0.096
≤60	199 (42.1)	2073 (47.1)	.04	191 (41.5)	213 (46.3)	0.096
BMI						
Mean (SD)	35.9 (7.8)	31.3 (7.4)	<.001	35.7 (7.6)	35.5 (8.8)	0.023
≤35	155 (32.8)	838 (19.1)	<.001	145 (31.5)	148 (32.2)	0.014
LDL Cholesterol, mg/dL						
Mean (SD)	83.2 (36.6)	83.1 (35.7)	.96	83.2 (36.7)	81.7 (39.5)	0.041
≥100	64 (13.5)	690 (15.7)	.22	63 (13.7)	61 (13.3)	0.013
Visit						
Inpatient encounter	75 (15.9)	695 (15.8)	.98	72 (15.7)	82 (17.8)	0.058
Emergency encounter	88 (18.6)	893 (20.3)	.38	85 (18.5)	78 (17.0)	0.040

Abbreviations: ACE, angiotensin-converting enzyme; BMI, body mass index (calculated as weight in kilograms divided by height in meters squared); DPP4, dipeptidyl peptidase-4; GLP-1, glucagon-like peptide-1; LDL, low-density lipoprotein; PS, propensity score; RA, receptor agonist; SGLT2, sodium-glucose co-transporter 2; SMD, standardized mean difference.

SI conversion factors: To convert cholesterol to millimoles per liter, multiply by 0.0259; hemoglobin A_1c_ to proportion of total hemoglobin, multiply by 0.01.

^a^ The following covariates were incorporated into the PS model: age, female, Black or African American, Hispanic or Latino, White, essential hypertension, chronic kidney disease, atrial fibrillation and flutter, morbid obesity, hyperlipidemia, β-blockers, ACE inhibitors, angiotensin II inhibitors, calcium channel blockers, statins, potassium sparing or combination diuretics, SGLT2 inhibitors, metformin, insulin, DPP4 inhibitors, sulfonylureas, thiazolidinediones, anticoagulants, aspirin, glomerular filtration rate, hemoglobin A_1c_, BMI, and LDL cholesterol.

The primary end point was MACCE, defined as a composite of all-cause mortality, new-onset HF, acute coronary syndrome, or any stroke or TIA. Secondary end points were individual components of the MACCE, decompensated HF, incident acute kidney injury (AKI) or progression to ESKD, and a composite of other CV events that were found to be associated with increased risk in MGUS^12^ (atrial fibrillation or flutter, conduction disorders, aortic or VHD, peripheral artery disease, arterial or VTE, and cor pulmonale). Given that DPP4Is also modulate the incretin pathway, albeit less potently than GLP-1 RAs, and SGLT2Is have established CV benefits, we performed sensitivity analyses by excluding patients with baseline use of DPP4Is or SGLT2Is to assess potential confounding. The detailed study end points are presented in eMethods 2 in Supplement 1.

We used 2 measures to test the residual confounders in the context of this observational study. First, we set 2 falsification end points: incident bone fracture and upper or lower respiratory infection. Second, we applied E-values^32^ to assess the potential impact of unmeasured confounders on the observed associations between GLP-1 RA use and the outcomes of interest. The E-value is a sensitivity analysis measure that quantifies the minimum strength of association that an unmeasured confounder would need to have with both GLP-1 RAs and study end points to fully explain away the observed association.^32^ Patients with baseline GLP-1 RA use prior to the diagnosis of MGUS continued to be classified as GLP-1 RA users, regardless of their discontinuation during the follow-up period. Similarly, those without baseline GLP-1 RA use continued to be considered nonusers irrespective of the start of use during the follow-up period. Cox proportional hazard ratios (HRs) were calculated to compare study end points between GLP-1 RA users and nonusers within 5 years of incident MGUS diagnosis in the matched cohort. Survival analyses for study end points were performed using Kaplan-Meier curves, with log-rank tests used to compare groups. Statistical significance was set at a 2-tailed *P* < .05. All analyses were performed using the TriNetX platform and RStudio software version 4.4.0 (R Project for Statistical Computing). Data analyses were completed January 19, 2025.

## Results

**Figure 1.**
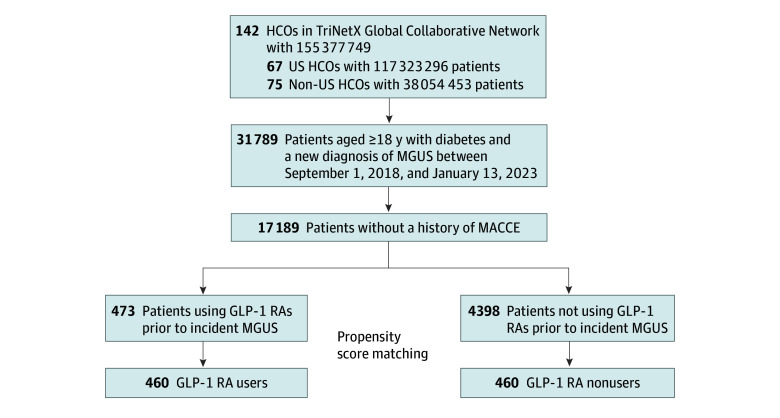
Patient Recruitment Flowchart Depicting Inclusion and Exclusion Criteria HCO indicates health care organizations.

A total of 4871 patients with MGUS (mean [SD] age, 68.9 [10.1] years; 2366 [48.5%] male) were included, with 473 GLP-1 RA users and 4398 nonusers (Figure 1). GLP-1 RA users, compared with nonusers, were younger (mean [SD] age, 64.7 [10.7] years vs 69.0 [10.8] years; *P* < .001), had similar numbers of male patients (232 [49.0%] male vs 2134 [48.5%] male; *P* = .52), had higher body mass index (BMI; calculated as weight in kilograms divided by height in meters squared) (mean [SD], 35.2 [7.4] vs 31.3 [7.2]; *P* < .001), and had more prevalent morbid obesity (87 patients [18.4%] vs 378 patients [8.6%]; *P* < .001) and history of lung cancers (10 patients [2.1%] vs 24 patients [0.5%]; *P* = .001). Hemoglobin A_1c_ was also higher in the GLP-1 RA group (mean [SD] 7.5% [1.7%] vs 7.1% [1.5%]; *P* < .001), with more prevalent use of other antidiabetic agents, including SGLT2I (90 patients [19.0%] vs 286 patients [6.5%]; *P* = .02), insulin (245 patients [51.8%] vs 1857 patients [42.2%]; *P* < .001), and thiazolidinediones (31 patients [6.6%] vs 183 patients [4.2%]; *P* < .001), except for metformin (232 patients [49.0%] vs 2487 patients [56.5%]; *P* = .002). After PS matching, 460 GLP-1 RA users were matched to 460 non-users **(**Figure 1**)**, with balanced baseline characteristics between GLP-1 RA users and nonusers (mean [SD] age, 65.0 [10.6] vs 65.1 [11.0] years; 229 [49.7%] male vs 234 [50.8%] male), including 14 patients (3.0%) vs 13 patients (2.8%) identifying as Asian, 8 (21.3%) vs 92 (20.0%) as Black or African American, 25 patients (5.4%) vs 20 patients (4.3%) as Hispanic or Latino, and 243 patients (52.8%) vs 250 patients (54.3%) as White (Table 1; eResults in Supplement 1).

### Study End Points

**Figure 2.**
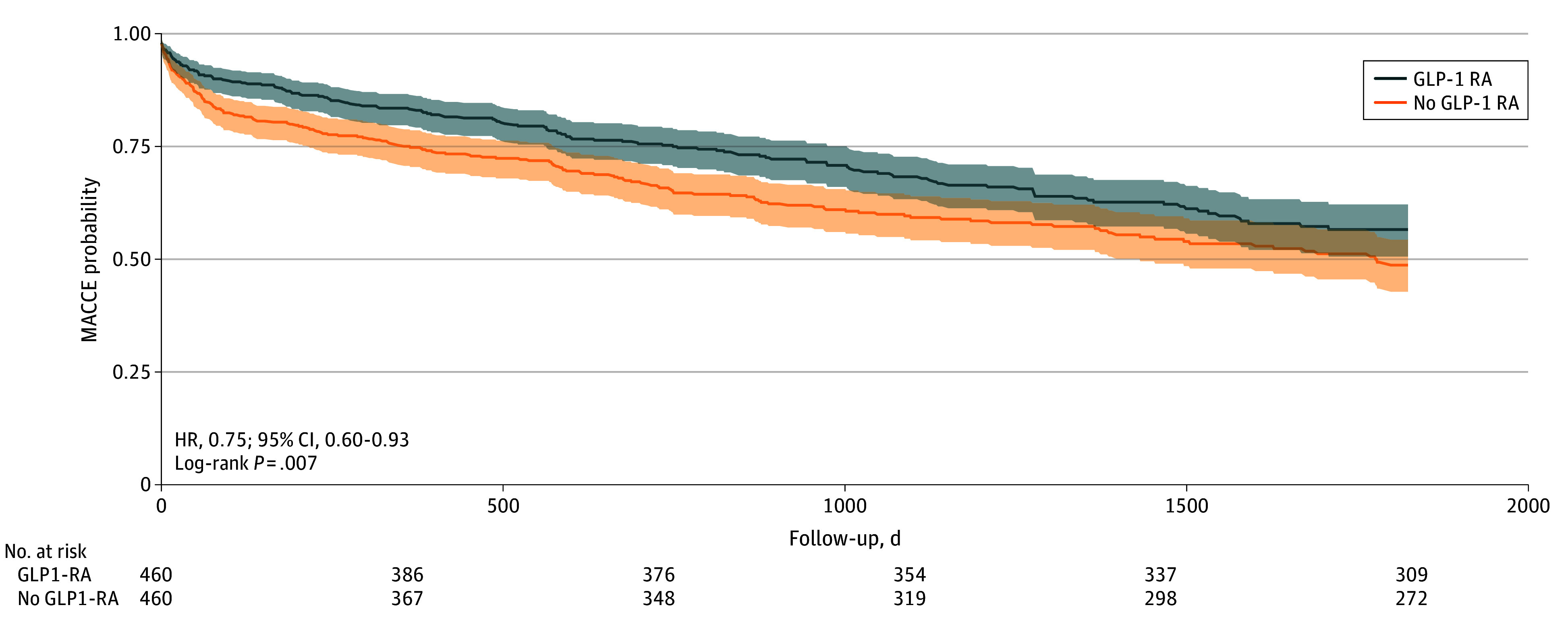
Kaplan-Meier Survival Curves for Comparative Risk of Major Adverse Cardiovascular and Cerebral Events (MACCE) Between Patients Using Glucagon-Like Peptide-1 (GLP-1) Receptor Inhibitors (RAs) and Those With No GLP-1 Use

Over the study follow-up period (median [IQR]: GLP-1 RA users, 3.2 [1.7-4.6] years; non-users, 3.2 [1.7-4.7] years), baseline GLP-1 RA use was associated with significantly lower 2-year MACCE (HR, 0.75; 95% CI, 0.60-0.93; *P* = .007; E-value = 2.00) (Figure 2) compared with nonuse. Secondary end point analysis revealed that GLP-1 RA use was associated with significant reductions in all-cause mortality (HR, 0.57; 95% CI, 0.37-0.82; *P* = .009; E-value = 2.90), new-onset HF (HR, 0.69; 95% CI, 0.54-0.90; *P* = .005; E-value = 2.26), decompensated HF (HR, 0.60; 95% CI, 0.43-0.84; *P* = .002; E-value = 2.72), AKI or progression to ESKD (HR, 0.73; 95% CI, 0.57-0.92; *P* = .01; E-value = 2.08), and other CV events (HR, 0.76; 95% CI, 0.62-0.93; *P* = .009; E-value = 1.96) (Table 2). The reductions in other CV events were primarily driven by cardiac arrhythmia (HR, 0.77; 95% CI, 0.59-1.00; *P* = .05), VHD (HR, 0.67; 95% CI, 0.48-0.94; *P* = .02), and cor pulmonale (HR, 0.63; 95% CI, 0.40-0.99; *P* = .04) (eTable 1 in Supplement 1). There was no significant difference in incident acute coronary syndrome (HR, 0.77; 95% CI, 0.46-1.28; *P* = .32) or stroke or TIA (HR, 0.75; 95% CI, 0.49-1.15; *P* = .19). Two falsification end points were comparable between the groups (Table 2). The sensitivity analysis excluding patients with baseline DPP4I or SGLT2I use showed a consistent association of GLP-1 RA use and significantly lower risk of MACCE (HR, 0.73; 95% CI, 0.57-0.95; *P* = .01) (eFigure in Supplement 1), all-cause mortality, new-onset HF, decompensated HF, AKI or progression to ESKD, and other CV events (eTable 2 in Supplement 1).

**Table 2.** Cox Proportional Hazard Analysis for the Association Between GLP-1 RA and Study End Points in the Matched Cohort

End point	Patients by GLP-1 RA use, No. (%)		HR (95% CI)	Log-rank *P* value	LNL E-value
	Users (n = 460)	Nonusers (n = 460)			
MACCE (primary end point)	151 (32.8)	188 (40.9)	0.75 (0.60-0.93)	.007	2.0 (2.72)
Secondary end points					
All-cause mortality	33 (7.2)	58 (12.6)	0.57 (0.37-0.82)	.009	2.9 (4.85)
New-onset HF	101 (22.0)	137 (29.8)	0.69 (0.54-0.90)	.005	2.26 (3.11)
Decompensated HF	59 (12.8)	92 (20.0)	0.60 (0.43-0.84)	.002	2.72 (4.08)
Incident stroke or TIA	38 (8.3)	49 (10.7)	0.75 (0.49-1.15)	.19	2.0 (3.5)
Incident ACS	27 (5.9)	34 (7.4)	0.77 (0.46-1.28)	.32	1.92 (3.77)
AKI or progression to ESKD	122 (26.5)	158 (34.3)	0.73 (0.57-0.92)	.01	2.08 (2.9)
Other CV events	176 (38.2)	213 (46.3)	0.76 (0.62-0.93)	.009	1.96 (2.61)
Falsification end points					
Respiratory infection	82 (17.8)	77 (16.7)	1.07 (0.79-1.47)	.63	NA
Fractures	47 (10.2)	52 (11.3)	0.91 (0.61-1.35)	.64	NA

Abbreviations: ACS, acute coronary syndrome; AKI, acute kidney injury; ESKD, end-stage kidney disease; GLP-1, glucagon-like peptide-1; HF, heart failure; HR, hazard ratio; LNL, lower normal limit; NA, not applicable; RA, receptor agonist; TIA, transient ischemic attack.

## Discussion

Our cohort study found that GLP-1 RA use was associated with a significant reduction in MACCE in patients with MGUS and diabetes compared with no use. The reduction in MACCE appeared to be primarily driven by decreases in all-cause mortality and new-onset HF. Additionally, GLP1-RA users experienced significantly lower risk of decompensated HF, AKI or progression to ESKD, and other CV events compared with nonusers. Given that patients with MGUS have reduced survival compared with the general population, likely due to their inherently higher burden of comorbidities, these findings suggest that the cardiac and renal benefits of GLP-1 RAs extend to patients with MGUS and diabetes. Unlike most existing evidence that demonstrated the CV benefits of GLP-1 RAs in patients with established MACCE or atherosclerotic CV disease, namely a secondary prevention approach,^33^ our study highlights the potential of GLP-1 RAs from a primary prevention perspective in an MGUS population. The potential of GLP-1 RAs to prevent incident HF and progression to ESKD is of paramount importance, as increasing evidence suggests that patients with MGUS are at higher risk of developing HF and kidney failure compared with the non-MGUS population.^12,34^ One potential mechanism involves paraprotein deposition in myocardial and renal tissues, manifesting monoclonal gammopathy of CV and renal significance. ^35,36^ Additionally, these processes are exacerbated by obesity, which is not only a recognized risk factor for MGUS^37^ but also plays a causal role in the progression to MM due to chronic inflammation characterized by dysregulated proinflammatory cytokines and adipokines associated with obesity.^38,39^ Of note, our study cohort had a mean BMI of more than 35, with up to 20% of patients classified as having morbid obesity. Given that emerging data indicate GLP-1 RA use may slow the progression of MGUS to MM,^40^ our findings further underscore a potential interaction between GLP-1 RAs and MGUS. However, these results should be interpreted cautiously, considering the cohort’s characteristics. More than one-third of patients with MGUS in our study had CKD, a prevalence markedly higher than the approximately 14% observed in the general US population.^41^ This elevated prevalence is likely attributable to the cohort’s high burden of obesity, a well-established risk factor for CKD.^42^ This characteristic carries clinical significance, as the intersection of obesity, diabetes, and CKD represents an established CV-kidney-metabolic (CKM) pathway in which GLP-1 RAs have consistently demonstrated CV and mortality benefits.^42^ For example, the FLOW trial^43^ found that GLP-1 RA use resulted in a significant reduction in CKD progression, MACCE, CV death, and all-cause mortality in patients with type 2 diabetes, CKD, and a mean BMI of 32. Additionally, the recently published SUMMIT trial^44^ demonstrated that GLP-1 RA use significantly reduced a composite of decompensated HF and CV death in patients with obesity and HF with preserved ejection fraction. Our findings, which demonstrated the benefits associated with GLP-1 RAs in reductions in incident HF, decompensated HF, and progression to ESKD, align with the outcome pattern of GLP-1 RAs identified in both the FLOW and SUMMIT trials. It is plausible that the observed protection against CV and renal events associated with GLP-1 RAs was mediated through the CKM pathway. However, it remains to be elucidated whether the observed benefits associated with GLP-1 RAs in this MGUS population involve a novel interaction with MGUS or are entirely mediated through established CKM mechanisms.

Our study builds on findings from the Danish National Patient Registry,^12^ which showed MGUS was associated with an elevated risk of a broad spectrum of CV comorbidities. The potential protective benefits associated with GLP-1 RAs reported in our study were primarily observed in new-onset HF, AKI or ESKD, VHD, cor pulmonale, and potential cardiac arrhythmia. Of note, the benefits observed in VHD, cor pulmonale, and cardiac arrhythmia may reflect a collinearity of improvements seen with reductions in new-onset HF, as cardiac remodeling in HF is linked to increased risk of these conditions, such as secondary mitral regurgitation and atrial fibrillation from increased ventricular filling.^45,46^ Conversely, there were no significant benefits in other end points, with several possible explanations. First, the sample size required to detect statistical significance may be larger for a primary than a secondary prevention design.^33^ This limitation arises because primary prevention populations, namely those without established MACCE or atherosclerotic CV disease, have fewer events than secondary prevention populations.^33^ Consequently, our analysis may have been underpowered to detect meaningful differences between GLP-1 RA users and nonusers across several neutral outcomes, particularly myocardial infarction, where prior trials have shown GLP-1 RA benefits.^33^ Second, it is likely not all the defined CV comorbidities are truly linked to MGUS and may instead reflect selection bias. In the absence of routine MGUS screening, the CV comorbidities identified in current literature may disproportionately represent patients with underlying conditions who underwent serum protein electrophoresis as a part of diagnostic work-ups.^47^ Thus, it remains uncertain whether the observed risk of CV comorbidities can be entirely attributed to MGUS, despite recognized inflammatory process and myocardial depositions of paraproteins. Third, it is possible that there is no direct interaction between GLP-1 RAs and MGUS. Instead, the observed benefits associated with GLP-1 RAs may be merely driven by CKM pathway in patients with obesity, diabetes, and CKD, as previously elaborated. Therefore, it is not surprising that no significant differences were observed in atrial fibrillation, aortic aneurysm, VHD, conduction disorders, peripheral artery disease, cor pulmonale, and venous thromboembolism events, since these events have not been established in prior CV outcome trials of GLP-1 RA.

### Limitations

Our study has several limitations. First, the observational design inherently limits causal inference. Second, the lack of individual patient data precludes analysis of GLP-1 RA dosage, titration, adherence, and potential treatment crossover, which may affect the precision of our effect estimates. Third, the high prevalence of obesity and elevated BMI in this cohort may limit the generalizability of our findings to patients with MGUS without obesity. Fourth, due to the constraints of the TriNetX Analytics Platform, we were unable to perform 1:2 or 1:3 propensity score matching, resulting in a relatively small matched sample size that precluded meaningful subgroup analyses. Fifth, our analysis shares the limitation of prior studies using TriNetX in its inability to account for matched-pair correlations, which may lead to overestimated effect sizes and artificially narrow CIs due to lack of individual-level data. Sixth, reliance on *ICD-10-CM* codes to define the study cohort and clinical end points introduces the risk of diagnostic misclassification. Furthermore, the dataset primarily includes patients from large academic centers, which may not fully represent broader or more diverse populations. Consequently, our findings require validation in other populations and through alternative data resources.

## Conclusions

In this cohort study of GLP-1 RA use vs no use in patients with MGUS and type 2 diabetes, our findings suggested the potential of GLP-1 RA for primary prevention of MACCE. Future prospective randomized clinical trials are warranted to validate these findings and further investigate the mechanisms underlying these observed benefits.

## Notes

Supplement 1. **eMethods 1.** Query Criteria for Study Cohort **eMethods 2.** Definitions for Study Endpoints **eResults.** Propensity-Score Matching in the Original Cohort **eTable 1.** Association Between GLP-1 RA and Other CV End Points in the Matched Cohort **eFigure.** Kaplan-Meier Survival Curves for Comparative Major Cardiac and Cerebrovascular Events in MGUS Patients With Type 2 Diabetes Treated With or Without Baseline GLP-1 RA. **eTable 2.** Sensitivity Analysis: Cox Proportional Hazard Analysis for the Association Between GLP-1 RA and Study End Points in the Cohort Excluding Patients With DPP4I and SGLT2I

Supplement 2.
Data Sharing Statement
