# Supplementary material for: GLP-1 RA Use and Major Adverse Cardiovascular Events in Patients With Monoclonal Gammopathy of Undetermined Significance
Source: JAMA Netw Open. 2025 Jun 30;8(6):e2517541. doi: 10.1001/jamanetworkopen.2025.17541 (PMC12210086; doi:10.1001/jamanetworkopen.2025.17541)
Supplement: Supplement 1. — eMethods 1. Query Criteria for Study Cohort eMethods 2. Definitions for Study Endpoints eResults. Propensity-Score Matching in the Original Cohort eTable 1. Association Between GLP-1 RA and Other CV End Points in the Matched Cohort eFigure. Kaplan-Meier Survival Curves for Comparative Major Cardiac and Cerebrovascular Events in MGUS Patients With Type 2 Diabetes Treated With or Without Baseline GLP-1 RA. eTable 2. Sensitivity Analysis: Cox Proportional Hazard Analysis for the Association Between GLP-1 RA and Study End Points in the Cohort Excluding Patients With DPP4I and SGLT2I [file jamanetwopen-e2517541-s001.pdf]

Supplemental Online Content

Chi KY, Song J, Desphande S, et al. GLP-1 RA use and major adverse cardiovascular events in patients with monoclonal gammopathy of undetermined significance. *JAMA Netw Open*. 2025;8(6):e2517541.
doi:10.1001/jamanetworkopen.2025.17541

eMethods 1. Query Criteria for Study Cohort
eMethods 2. Definitions for Study Endpoints
eResults. Propensity-Score Matching in the Original Cohort
eTable 1. Association Between GLP-1 RA and Other CV End Points in the Matched Cohort
eFigure. Kaplan-Meier Survival Curves for Comparative Major Cardiac and Cerebrovascular Events in MGUS Patients With Type 2 Diabetes Treated With or Without Baseline GLP-1 RA.
eTable 2. Sensitivity Analysis: Cox Proportional Hazard Analysis for the Association Between GLP-1 RA and Study End Points in the Cohort Excluding Patients With DPP4I and SGLT2I

This supplemental material has been provided by the authors to give readers additional information about their work.

© 2025 Chi KY et al. *JAMA Network Open*.

Supplemental Methods 1. Query Criteria for Study Cohort

Query Criteria for Cohort 1 (query name: GLP-1 RA users)

This query was run on the network Global Collaborative Network with 143 HCO(s) queried and 143 HCO(s) responded. A total of 49 provider(s) responded with patients. The final cohort included 473 patients who matched the query criteria listed in the table below. For the text representation of the query criteria please see Appendix A.

**Group 1**				
**Group 1A**				
must have		diagnosis	UMLS:ICD10CM:D47.2	Monoclonal gammopathy
	and	diagnosis	UMLS:ICD10CM:E11	Type 2 diabetes mellitus
date constraint		The terms in this group occurred between Jan 1, 2018 and Jan 13, 2023		
event relationship		Any instance of Group 1B occurred on or before the first instance of Group 1A		
**Group 1B**				
cannot have		diagnosis	UMLS:ICD10CM:C90.0	Multiple myeloma
	or	laboratory	UMLS:LNC:11118-7	Plasma cells/100 cells in Bone marrow by Manual count (at least 10.00 %)
	or	laboratory	UMLS:LNC:33358-3	Protein.monoclonal [Mass/volume] in Serum or Plasma by Electrophoresis (at least 3.00 g/dL)
	or	diagnosis	UMLS:ICD10CM:D89.0	Polyclonal hypergammaglobulinemia
	or	diagnosis	UMLS:ICD10CM:E85	Amyloidosis
	or	demographics	Deceased	Deceased
	or	diagnosis	UMLS:ICD10CM:I50	Heart failure
	or	diagnosis	UMLS:ICD10CM:I42	Cardiomyopathy
	or	laboratory	TNX:FINDING:2003	Left Ventricular Ejection Fraction (LVEF) (%) (at most 50.00 %)
	or	diagnosis	UMLS:ICD10CM:I60	Nontraumatic subarachnoid hemorrhage
	or	diagnosis	UMLS:ICD10CM:I61	Nontraumatic intracerebral hemorrhage
	or	diagnosis	UMLS:ICD10CM:I62	Other and unspecified nontraumatic intracranial hemorrhage
	or	diagnosis	UMLS:ICD10CM:I63	Cerebral infarction
	or	diagnosis	UMLS:ICD10CM:G45	Transient cerebral ischemic attacks and related syndromes
	or	diagnosis	UMLS:ICD10CM:I67.82	Cerebral ischemia
	or	procedure	UMLS:CPT:92920	Percutaneous transluminal coronary angioplasty; single major coronary artery or branch
	or	procedure	UMLS:CPT:92928	Percutaneous transcatheter placement of intracoronary stent(s), with coronary angioplasty when performed; single major coronary artery or branch
	or	procedure	UMLS:CPT:92929	Percutaneous transcatheter placement of intracoronary stent(s), with coronary angioplasty when performed; each additional branch of a major coronary artery (List

© 2025 Chi KY et al. *JAMA Network Open*.

			separately in addition to code for primary procedure)
or	procedure	UMLS:CPT:92921	Percutaneous transluminal coronary angioplasty; each additional branch of a major coronary artery (List separately in addition to code for primary procedure)
or	procedure	UMLS:CPT:92924	Percutaneous transluminal coronary atherectomy, with coronary angioplasty when performed; single major coronary artery or branch
or	procedure	UMLS:CPT:92925	Percutaneous transluminal coronary atherectomy, with coronary angioplasty when performed; each additional branch of a major coronary artery (List separately in addition to code for primary procedure)
or	procedure	UMLS:CPT:92933	Percutaneous transluminal coronary atherectomy, with intracoronary stent, with coronary angioplasty when performed; single major coronary artery or branch
or	procedure	UMLS:CPT:92934	Percutaneous transluminal coronary atherectomy, with intracoronary stent, with coronary angioplasty when performed; each additional branch of a major coronary artery (List separately in addition to code for primary procedure)
or	procedure	UMLS:CPT:92937	Percutaneous transluminal revascularization of or through coronary artery bypass graft (internal mammary, free arterial, venous), any combination of intracoronary stent, atherectomy and angioplasty, including distal protection when performed; single vessel
or	procedure	UMLS:CPT:92938	Percutaneous transluminal revascularization of or through coronary artery bypass graft (internal mammary, free arterial, venous), any combination of intracoronary stent, atherectomy and angioplasty, including distal protection when performed; each additional branch subtended by the bypass graft (List separately in addition to code for primary procedure)
or	procedure	UMLS:CPT:92941	Percutaneous transluminal revascularization of acute total/subtotal occlusion during acute myocardial infarction, coronary artery or coronary artery bypass graft, any combination of intracoronary stent, atherectomy and angioplasty, including aspiration

			thrombectomy when performed, single vessel
or	procedure	UMLS:CPT:92943	Percutaneous transluminal revascularization of chronic total occlusion, coronary artery, coronary artery branch, or coronary artery bypass graft, any combination of intracoronary stent, atherectomy and angioplasty; single vessel
or	procedure	UMLS:CPT:92944	Percutaneous transluminal revascularization of chronic total occlusion, coronary artery, coronary artery branch, or coronary artery bypass graft, any combination of intracoronary stent, atherectomy and angioplasty; each additional coronary artery, coronary artery branch, or bypass graft (List separately in addition to code for primary procedure)
or	procedure	UMLS:CPT:92978	Endoluminal imaging of coronary vessel or graft using intravascular ultrasound (IVUS) or optical coherence tomography (OCT) during diagnostic evaluation and/or therapeutic intervention including imaging supervision, interpretation and report; initial vessel (List separately in addition to code for primary procedure)
or	procedure	UMLS:CPT:92979	Endoluminal imaging of coronary vessel or graft using intravascular ultrasound (IVUS) or optical coherence tomography (OCT) during diagnostic evaluation and/or therapeutic intervention including imaging supervision, interpretation and report; each additional vessel (List separately in addition to code for primary procedure)
or	procedure	UMLS:CPT:92973	Percutaneous transluminal coronary thrombectomy mechanical (List separately in addition to code for primary procedure)
or	procedure	UMLS:CPT:92977	Thrombolysis, coronary; by intravenous infusion
or	procedure	UMLS:CPT:92974	Transcatheter placement of radiation delivery device for subsequent coronary intravascular brachytherapy (List separately in addition to code for primary procedure)
or	procedure	UMLS:CPT:92975	Thrombolysis, coronary; by intracoronary infusion, including selective coronary angiography
or	procedure	UMLS:CPT:93571	Intravascular Doppler velocity and/or pressure derived coronary flow reserve measurement (coronary

			vessel or graft) during coronary angiography including pharmacologically induced stress; initial vessel (List separately in addition to code for primary procedure)
or	procedure	UMLS:CPT:93572	Intravascular Doppler velocity and/or pressure derived coronary flow reserve measurement (coronary vessel or graft) during coronary angiography including pharmacologically induced stress; each additional vessel (List separately in addition to code for primary procedure)
or	procedure	UMLS:HCPCS:C9600	Percutaneous transcatheter placement of drug eluting intracoronary stent(s), with coronary angioplasty when performed; single major coronary artery or branch
or	procedure	UMLS:HCPCS:C9606	Percutaneous transluminal revascularization of acute total/subtotal occlusion during acute myocardial infarction, coronary artery or coronary artery bypass graft, any combination of drug-eluting intracoronary stent, atherectomy and angioplasty, including aspiration thrombectomy when performed, single vessel
or	procedure	UMLS:HCPCS:C9601	Percutaneous transcatheter placement of drug-eluting intracoronary stent(s), with coronary angioplasty when performed; each additional branch of a major coronary artery (list separately in addition to code for primary procedure)
or	procedure	UMLS:HCPCS:C9602	Percutaneous transluminal coronary atherectomy, with drug eluting intracoronary stent, with coronary angioplasty when performed; single major coronary artery or branch
or	procedure	UMLS:HCPCS:C9604	Percutaneous transluminal revascularization of or through coronary artery bypass graft (internal mammary, free arterial, venous), any combination of drug-eluting intracoronary stent, atherectomy and angioplasty, including distal protection when performed; single vessel
or	procedure	UMLS:HCPCS:C9607	Percutaneous transluminal revascularization of chronic total occlusion, coronary artery, coronary artery branch, or coronary artery bypass graft, any combination of drug-eluting intracoronary stent,

© 2025 Chi KY et al. *JAMA Network Open*.

				atherectomy and angioplasty; single vessel
	or	procedure	UMLS:HCPCS:C9603	Percutaneous transluminal coronary atherectomy, with drug-eluting intracoronary stent, with coronary angioplasty when performed; each additional branch of a major coronary artery (list separately in addition to code for primary procedure)
	or	procedure	UMLS:HCPCS:C9605	Percutaneous transluminal revascularization of or through coronary artery bypass graft (internal mammary, free arterial, venous), any combination of drug-eluting intracoronary stent, atherectomy and angioplasty, including distal protection when performed; each additional branch subtended by the bypass graft (list separately in addition to code for primary procedure)
	or	procedure	UMLS:HCPCS:C9608	Percutaneous transluminal revascularization of chronic total occlusion, coronary artery, coronary artery branch, or coronary artery bypass graft, any combination of drug-eluting intracoronary stent, atherectomy and angioplasty; each additional coronary artery, coronary artery branch, or bypass graft (list separately in addition to code for primary procedure)
	or	diagnosis	UMLS:ICD10CM:I20-I25	Ischemic heart diseases
	or	diagnosis	UMLS:ICD10CM:I43	Cardiomyopathy in diseases classified elsewhere
**Group 2**				
**Group 2A**				
must have		diagnosis	UMLS:ICD10CM:E11	Type 2 diabetes mellitus
	and	diagnosis	UMLS:ICD10CM:D47.2	Monoclonal gammopathy
date constraint		The terms in this group occurred between Jan 1, 2018 and Jan 13, 2023		
event relationship		Any instance of Group 2B occurred within 1 year on or before the first instance of Group 2A		
**Group 2B**				
must have	any of	medication	NLM:ATC:A10BJ	Glucagon-like peptide-1 (GLP-1) analogues
		medication	NLM:RXNORM:2601723	tirzepatide

Query Criteria for Cohort 2 (query name: GLP1-RA non-users)

This query was run on the network Global Collaborative Network with 143 HCO(s) queried and 143 HCO(s) responded. A total of 63 provider(s) responded with patients. The final cohort included 4,398 patients who matched the query criteria listed in the table below.

**Group 1**	
**Group 1A**	

© 2025 Chi KY et al. *JAMA Network Open*.

must have		diagnosis	UMLS:ICD10CM:D47.2	Monoclonal gammopathy
	and	diagnosis	UMLS:ICD10CM:E11	Type 2 diabetes mellitus
date constraint		The terms in this group occurred between Jan 1, 2018 and Jan 13, 2023		
event relationship		Any instance of Group 1B occurred on or before the first instance of Group 1A		
**Group 1B**				
cannot have		diagnosis	UMLS:ICD10CM:C90.0	Multiple myeloma
	or	laboratory	UMLS:LNC:11118-7	Plasma cells/100 cells in Bone marrow by Manual count (at least 10.00 %)
	or	laboratory	UMLS:LNC:33358-3	Protein.monoclonal [Mass/volume] in Serum or Plasma by Electrophoresis (at least 3.00 g/dL)
	or	diagnosis	UMLS:ICD10CM:D89.0	Polyclonal hypergammaglobulinemia
	or	diagnosis	UMLS:ICD10CM:E85	Amyloidosis
	or	demographics	Deceased	Deceased
	or	diagnosis	UMLS:ICD10CM:I50	Heart failure
	or	diagnosis	UMLS:ICD10CM:I42	Cardiomyopathy
	or	laboratory	TNX:FINDING:2003	Left Ventricular Ejection Fraction (LVEF) (%) (at most 50.00 %)
	or	diagnosis	UMLS:ICD10CM:I60	Nontraumatic subarachnoid hemorrhage
	or	diagnosis	UMLS:ICD10CM:I61	Nontraumatic intracerebral hemorrhage
	or	diagnosis	UMLS:ICD10CM:I62	Other and unspecified nontraumatic intracranial hemorrhage
	or	diagnosis	UMLS:ICD10CM:I63	Cerebral infarction
	or	diagnosis	UMLS:ICD10CM:I67.82	Cerebral ischemia
	or	procedure	UMLS:CPT:92928	Percutaneous transcatheter placement of intracoronary stent(s), with coronary angioplasty when performed; single major coronary artery or branch
	or	procedure	UMLS:CPT:92920	Percutaneous transluminal coronary angioplasty; single major coronary artery or branch
	or	procedure	UMLS:CPT:92929	Percutaneous transcatheter placement of intracoronary stent(s), with coronary angioplasty when performed; each additional branch of a major coronary artery (List separately in addition to code for primary procedure)
	or	procedure	UMLS:CPT:92921	Percutaneous transluminal coronary angioplasty; each additional branch of a major coronary artery (List separately in addition to code for primary procedure)
	or	procedure	UMLS:CPT:92924	Percutaneous transluminal coronary atherectomy, with coronary angioplasty when performed; single major coronary artery or branch
	or	procedure	UMLS:CPT:92925	Percutaneous transluminal coronary atherectomy, with coronary angioplasty when performed; each additional branch of a major coronary artery (List separately in

© 2025 Chi KY et al. *JAMA Network Open*.

			addition to code for primary procedure)
or	procedure	UMLS:CPT:92933	Percutaneous transluminal coronary atherectomy, with intracoronary stent, with coronary angioplasty when performed; single major coronary artery or branch
or	procedure	UMLS:CPT:92937	Percutaneous transluminal revascularization of or through coronary artery bypass graft (internal mammary, free arterial, venous), any combination of intracoronary stent, atherectomy and angioplasty, including distal protection when performed; single vessel
or	procedure	UMLS:CPT:92934	Percutaneous transluminal coronary atherectomy, with intracoronary stent, with coronary angioplasty when performed; each additional branch of a major coronary artery (List separately in addition to code for primary procedure)
or	procedure	UMLS:CPT:92938	Percutaneous transluminal revascularization of or through coronary artery bypass graft (internal mammary, free arterial, venous), any combination of intracoronary stent, atherectomy and angioplasty, including distal protection when performed; each additional branch subtended by the bypass graft (List separately in addition to code for primary procedure)
or	procedure	UMLS:CPT:92941	Percutaneous transluminal revascularization of acute total/subtotal occlusion during acute myocardial infarction, coronary artery or coronary artery bypass graft, any combination of intracoronary stent, atherectomy and angioplasty, including aspiration thrombectomy when performed, single vessel
or	procedure	UMLS:CPT:92943	Percutaneous transluminal revascularization of chronic total occlusion, coronary artery, coronary artery branch, or coronary artery bypass graft, any combination of intracoronary stent, atherectomy and angioplasty; single vessel
or	procedure	UMLS:CPT:92944	Percutaneous transluminal revascularization of chronic total occlusion, coronary artery, coronary artery branch, or coronary artery bypass graft, any combination of intracoronary stent, atherectomy and angioplasty; each additional coronary artery, coronary artery

			branch, or bypass graft (List separately in addition to code for primary procedure)
or	procedure	UMLS:CPT:92978	Endoluminal imaging of coronary vessel or graft using intravascular ultrasound (IVUS) or optical coherence tomography (OCT) during diagnostic evaluation and/or therapeutic intervention including imaging supervision, interpretation and report; initial vessel (List separately in addition to code for primary procedure)
or	procedure	UMLS:CPT:92979	Endoluminal imaging of coronary vessel or graft using intravascular ultrasound (IVUS) or optical coherence tomography (OCT) during diagnostic evaluation and/or therapeutic intervention including imaging supervision, interpretation and report; each additional vessel (List separately in addition to code for primary procedure)
or	procedure	UMLS:CPT:92973	Percutaneous transluminal coronary thrombectomy mechanical (List separately in addition to code for primary procedure)
or	procedure	UMLS:CPT:92977	Thrombolysis, coronary; by intravenous infusion
or	procedure	UMLS:CPT:92974	Transcatheter placement of radiation delivery device for subsequent coronary intravascular brachytherapy (List separately in addition to code for primary procedure)
or	procedure	UMLS:CPT:92975	Thrombolysis, coronary; by intracoronary infusion, including selective coronary angiography
or	procedure	UMLS:CPT:93571	Intravascular Doppler velocity and/or pressure derived coronary flow reserve measurement (coronary vessel or graft) during coronary angiography including pharmacologically induced stress; initial vessel (List separately in addition to code for primary procedure)
or	procedure	UMLS:CPT:93571	Intravascular Doppler velocity and/or pressure derived coronary flow reserve measurement (coronary vessel or graft) during coronary angiography including pharmacologically induced stress; initial vessel (List separately in addition to code for primary procedure)
or	procedure	UMLS:CPT:93572	Intravascular Doppler velocity and/or pressure derived coronary flow

© 2025 Chi KY et al. *JAMA Network Open*.

			reserve measurement (coronary vessel or graft) during coronary angiography including pharmacologically induced stress; each additional vessel (List separately in addition to code for primary procedure)
or	procedure	UMLS:HCPCS:C9600	Percutaneous transcatheter placement of drug eluting intracoronary stent(s), with coronary angioplasty when performed; single major coronary artery or branch
or	procedure	UMLS:HCPCS:C9606	Percutaneous transluminal revascularization of acute total/subtotal occlusion during acute myocardial infarction, coronary artery or coronary artery bypass graft, any combination of drug-eluting intracoronary stent, atherectomy and angioplasty, including aspiration thrombectomy when performed, single vessel
or	procedure	UMLS:HCPCS:C9601	Percutaneous transcatheter placement of drug-eluting intracoronary stent(s), with coronary angioplasty when performed; each additional branch of a major coronary artery (list separately in addition to code for primary procedure)
or	procedure	UMLS:HCPCS:C9602	Percutaneous transluminal coronary atherectomy, with drug eluting intracoronary stent, with coronary angioplasty when performed; single major coronary artery or branch
or	procedure	UMLS:HCPCS:C9604	Percutaneous transluminal revascularization of or through coronary artery bypass graft (internal mammary, free arterial, venous), any combination of drug-eluting intracoronary stent, atherectomy and angioplasty, including distal protection when performed; single vessel
or	procedure	UMLS:HCPCS:C9607	Percutaneous transluminal revascularization of chronic total occlusion, coronary artery, coronary artery branch, or coronary artery bypass graft, any combination of drug-eluting intracoronary stent, atherectomy and angioplasty; single vessel
or	procedure	UMLS:HCPCS:C9603	Percutaneous transluminal coronary atherectomy, with drug-eluting intracoronary stent, with coronary angioplasty when performed; each additional branch of a major coronary artery (list separately in

© 2025 Chi KY et al. *JAMA Network Open*.

				addition to code for primary procedure)
	or	procedure	UMLS:HCPCS:C9605	Percutaneous transluminal revascularization of or through coronary artery bypass graft (internal mammary, free arterial, venous), any combination of drug-eluting intracoronary stent, atherectomy and angioplasty, including distal protection when performed; each additional branch subtended by the bypass graft (list separately in addition to code for primary procedure)
	or	procedure	UMLS:HCPCS:C9608	Percutaneous transluminal revascularization of chronic total occlusion, coronary artery, coronary artery branch, or coronary artery bypass graft, any combination of drug-eluting intracoronary stent, atherectomy and angioplasty; each additional coronary artery, coronary artery branch, or bypass graft (list separately in addition to code for primary procedure)
	or	diagnosis	UMLS:ICD10CM:I20-I25	Ischemic heart diseases
	or	diagnosis	UMLS:ICD10CM:I43	Cardiomyopathy in diseases classified elsewhere
**Group 2**				
**Group 2A**				
must have		diagnosis	UMLS:ICD10CM:E11	Type 2 diabetes mellitus
	and	diagnosis	UMLS:ICD10CM:D47.2	Monoclonal gammopathy
date constraint		The terms in this group occurred between Jan 1, 2018 and Jan 13, 2023		
event relationship		Any instance of Group 2B occurred within 1 year on or before the first instance of Group 2A		
**Group 2B**				
must have	any of	medication	NLM:ATC:A10BK	Sodium-glucose co-transporter 2 (SGLT2) inhibitors
		medication	NLM:ATC:A10BF	Alpha glucosidase inhibitors
		medication	NLM:ATC:A10BB	Sulfonylureas
		medication	NLM:VA:HS501	INSULIN
		medication	NLM:ATC:A10BH	Dipeptidyl peptidase 4 (DPP-4) inhibitors
		medication	NLM:RXNORM:6809	metformin
		medication	NLM:ATC:A10BG	Thiazolidinediones
cannot have		medication	NLM:ATC:A10BJ	Glucagon-like peptide-1 (GLP-1) analogues
	or	medication	NLM:RXNORM:2601723	tirzepatide
**Group 3**				
**Group 3A**				
must have		diagnosis	UMLS:ICD10CM:D47.2	Monoclonal gammopathy
date constraint		The terms in this group occurred between Jan 1, 2018 and Jan 13, 2023		
event relationship		Any instance of Group 3B occurred on or before the first instance of Group 3A		
**Group 3B**				
cannot have		medication	NLM:ATC:A10BJ	Glucagon-like peptide-1 (GLP-1) analogues
	or	medication	NLM:RXNORM:2601723	tirzepatide

© 2025 Chi KY et al. *JAMA Network Open*.

**Group 4**				
**Group 4A**				
must have		diagnosis	UMLS:ICD10CM:E11	Type 2 diabetes mellitus
	and	diagnosis	UMLS:ICD10CM:D47.2	Monoclonal gammopathy
date constraint		The terms in this group occurred between Jan 1, 2018 and Jan 13, 2023		
event relationship		Any instance of Group 4B occurred within 6 months on or before the first instance of Group 4A		
**Group 4B**				
cannot have		visit	UMLS:HL7V3.0:VisitType:IMP	Visit: Inpatient Encounter

© 2025 Chi KY et al. *JAMA Network Open*.

Supplemental Methods 2. Definitions for Study Endpoints

Outcome Definitions

Table below outlines the definitions for each outcome and the analysis specifications. For outcome definitions consisting of more than one term, at least one term must match.

**MACCE**			
**Outcome definition**			
	Demographics	Deceased	Deceased
	Diagnosis	UMLS:ICD10CM:I21	Acute myocardial infarction
	Diagnosis	UMLS:ICD10CM:I22	Subsequent ST elevation (STEMI) and non-ST elevation (NSTEMI) myocardial infarction
	Diagnosis	UMLS:ICD10CM:I63	Cerebral infarction
	Diagnosis	UMLS:ICD10CM:I67.82	Cerebral ischemia
	Diagnosis	UMLS:ICD10CM:I60	Nontraumatic subarachnoid hemorrhage
	Diagnosis	UMLS:ICD10CM:I61	Nontraumatic intracerebral hemorrhage
	Diagnosis	UMLS:ICD10CM:I62	Other and unspecified nontraumatic intracranial hemorrhage
	Diagnosis	UMLS:ICD10CM:I50	Heart failure
	Diagnosis	UMLS:ICD10CM:I42	Cardiomyopathy
	Laboratory	TNX:FINDING:2003	Left Ventricular Ejection Fraction (LVEF) (%) (at most 50.00 % (most recent occurrence))
	Diagnosis	UMLS:ICD10CM:G45	Transient cerebral ischemic attacks and related syndromes
	Diagnosis	UMLS:ICD10CM:I20.0	Unstable angina
	Diagnosis	UMLS:ICD10CM:I43	Cardiomyopathy in diseases classified elsewhere
	Medication	NLM:ATC:C03	DIURETICS (Route: Injectable Product)
**Death**			
**Outcome definition**			
	Demographics	Deceased	Deceased
**New-onset heart failure**			
**Outcome definition**			
	Diagnosis	UMLS:ICD10CM:I50	Heart failure
	Diagnosis	UMLS:ICD10CM:I42	Cardiomyopathy
	Laboratory	TNX:FINDING:2003	Left Ventricular Ejection Fraction (LVEF) (%) (at most 50.00 % (most recent occurrence))
	Diagnosis	UMLS:ICD10CM:I43	Cardiomyopathy in diseases classified elsewhere
	Medication	NLM:ATC:C03	DIURETICS (Route: Injectable Product)
**ACS**			
**Outcome definition**			
	Diagnosis	UMLS:ICD10CM:I21	Acute myocardial infarction
	Diagnosis	UMLS:ICD10CM:I22	Subsequent ST elevation (STEMI) and non-ST elevation (NSTEMI) myocardial infarction
	Diagnosis	UMLS:ICD10CM:I20.0	Unstable angina
**Decompensated heart failure**			
**Outcome definition**			
	Diagnosis	UMLS:ICD10CM:I50.33	Acute on chronic diastolic (congestive) heart failure
	Diagnosis	UMLS:ICD10CM:I50.23	Acute on chronic systolic (congestive) heart failure
	Diagnosis	UMLS:ICD10CM:I50.21	Acute systolic (congestive) heart failure
	Diagnosis	UMLS:ICD10CM:I50.43	Acute on chronic combined systolic (congestive) and diastolic (congestive) heart failure
	Diagnosis	UMLS:ICD10CM:I50.31	Acute diastolic (congestive) heart failure
	Diagnosis	UMLS:ICD10CM:I50.41	Acute combined systolic (congestive) and diastolic (congestive) heart failure
	Medication	NLM:ATC:C03	DIURETICS (Route: Injectable Product)

© 2025 Chi KY et al. *JAMA Network Open*.

**Any stroke or TIA**		
**Outcome definition**		
Diagnosis	UMLS:ICD10CM:I63	Cerebral infarction
Diagnosis	UMLS:ICD10CM:G45	Transient cerebral ischemic attacks and related syndromes
Diagnosis	UMLS:ICD10CM:I67.82	Cerebral ischemia
Diagnosis	UMLS:ICD10CM:I60	Nontraumatic subarachnoid hemorrhage
Diagnosis	UMLS:ICD10CM:I61	Nontraumatic intracerebral hemorrhage
Diagnosis	UMLS:ICD10CM:I62	Other and unspecified nontraumatic intracranial hemorrhage
**Other CV endpoints**		
**Outcome definition**		
Diagnosis	UMLS:ICD10CM:I46	Cardiac arrest
Diagnosis	UMLS:ICD10CM:I47	Paroxysmal tachycardia
Diagnosis	UMLS:ICD10CM:I48	Atrial fibrillation and flutter
Diagnosis	UMLS:ICD10CM:I49	Other cardiac arrhythmias
Diagnosis	UMLS:ICD10CM:I26	Pulmonary embolism
Diagnosis	UMLS:ICD10CM:I82.210	Acute embolism and thrombosis of superior vena cava
Diagnosis	UMLS:ICD10CM:I82.220	Acute embolism and thrombosis of inferior vena cava
Diagnosis	UMLS:ICD10CM:I82.290	Acute embolism and thrombosis of other thoracic veins
Diagnosis	UMLS:ICD10CM:I82.4	Acute embolism and thrombosis of deep veins of lower extremity
Diagnosis	UMLS:ICD10CM:I82.6	Acute embolism and thrombosis of veins of upper extremity
Diagnosis	UMLS:ICD10CM:I82.890	Acute embolism and thrombosis of other specified veins
Diagnosis	UMLS:ICD10CM:I82.90	Acute embolism and thrombosis of unspecified vein
Diagnosis	UMLS:ICD10CM:I74	Arterial embolism and thrombosis
Diagnosis	UMLS:ICD10CM:I75	Atheroembolism
Diagnosis	UMLS:ICD10CM:I80	Phlebitis and thrombophlebitis
Diagnosis	UMLS:ICD10CM:I81	Portal vein thrombosis
Diagnosis	UMLS:ICD10CM:I34	Nonrheumatic mitral valve disorders
Diagnosis	UMLS:ICD10CM:I35	Nonrheumatic aortic valve disorders
Diagnosis	UMLS:ICD10CM:I36	Nonrheumatic tricuspid valve disorders
Diagnosis	UMLS:ICD10CM:I37	Nonrheumatic pulmonary valve disorders
Diagnosis	UMLS:ICD10CM:I71	Aortic aneurysm and dissection
Diagnosis	UMLS:ICD10CM:I44	Atrioventricular and left bundle-branch block
Diagnosis	UMLS:ICD10CM:I45	Other conduction disorders
Procedure	UMLS:CPT:1006075	Pacemaker or Implantable Defibrillator Procedures
Diagnosis	UMLS:ICD10CM:I27	Other pulmonary heart diseases
Diagnosis	UMLS:ICD10CM:I73	Other peripheral vascular diseases
**Falsification 1: Respiratory infection**		
**Outcome definition**		
Diagnosis	UMLS:ICD10CM:J00-J06	Acute upper respiratory infections
**Falsification 2: fracture**		
**Outcome definition**		
Diagnosis	UMLS:ICD10CM:S02	Fracture of skull and facial bones
Diagnosis	UMLS:ICD10CM:S22	Fracture of rib(s), sternum and thoracic spine
Diagnosis	UMLS:ICD10CM:S42	Fracture of shoulder and upper arm
Diagnosis	UMLS:ICD10CM:S52	Fracture of forearm
Diagnosis	UMLS:ICD10CM:S72	Fracture of femur
Diagnosis	UMLS:ICD10CM:S82	Fracture of lower leg, including ankle
Diagnosis	UMLS:ICD10CM:S62	Fracture at wrist and hand level
Diagnosis	UMLS:ICD10CM:S92	Fracture of foot and toe, except ankle
Diagnosis	UMLS:ICD10CM:S12	Fracture of cervical vertebra and other parts of neck
Diagnosis	UMLS:ICD10CM:S32	Fracture of lumbar spine and pelvis
Diagnosis	UMLS:ICD10CM:S62	Fracture at wrist and hand level
**Cardiac arrhythmia**		
**Outcome definition**		
Diagnosis	UMLS:ICD10CM:I48	Atrial fibrillation and flutter

© 2025 Chi KY et al. *JAMA Network Open*.

Diagnosis	UMLS:ICD10CM:I46	Cardiac arrest
Diagnosis	UMLS:ICD10CM:I47	Paroxysmal tachycardia
Diagnosis	UMLS:ICD10CM:I49	Other cardiac arrhythmias
**Arterial and VTE**		
**Outcome definition**		
Diagnosis	UMLS:ICD10CM:I26	Pulmonary embolism
Diagnosis	UMLS:ICD10CM:I82.6	Acute embolism and thrombosis of veins of upper extremity
Diagnosis	UMLS:ICD10CM:I82.4	Acute embolism and thrombosis of deep veins of lower extremity
Diagnosis	UMLS:ICD10CM:I82.890	Acute embolism and thrombosis of other specified veins
Diagnosis	UMLS:ICD10CM:I82.90	Acute embolism and thrombosis of unspecified vein
Diagnosis	UMLS:ICD10CM:I82.220	Acute embolism and thrombosis of inferior vena cava
Diagnosis	UMLS:ICD10CM:I82.290	Acute embolism and thrombosis of other thoracic veins
Diagnosis	UMLS:ICD10CM:I82.210	Acute embolism and thrombosis of superior vena cava
Diagnosis	UMLS:ICD10CM:I74	Arterial embolism and thrombosis
Diagnosis	UMLS:ICD10CM:I75	Atheroembolism
Diagnosis	UMLS:ICD10CM:I80	Phlebitis and thrombophlebitis
Diagnosis	UMLS:ICD10CM:I81	Portal vein thrombosis
**Aortic or valvular heart disease**		
**Outcome definition**		
Diagnosis	UMLS:ICD10CM:I34	Nonrheumatic mitral valve disorders
Diagnosis	UMLS:ICD10CM:I35	Nonrheumatic aortic valve disorders
Diagnosis	UMLS:ICD10CM:I36	Nonrheumatic tricuspid valve disorders
Diagnosis	UMLS:ICD10CM:I37	Nonrheumatic pulmonary valve disorders
Diagnosis	UMLS:ICD10CM:I71	Aortic aneurysm and dissection
**Conduction disorder**		
**Outcome definition**		
Diagnosis	UMLS:ICD10CM:I44	Atrioventricular and left bundle-branch block
Diagnosis	UMLS:ICD10CM:I45	Other conduction disorders
Procedure	UMLS:CPT:1006075	Pacemaker or Implantable Defibrillator Procedures
**Cor pulmonale**		
**Outcome definition**		
Diagnosis	UMLS:ICD10CM:I27	Other pulmonary heart diseases
**PAD**		
**Outcome definition**		
Diagnosis	UMLS:ICD10CM:I73	Other peripheral vascular diseases

© 2025 Chi KY et al. *JAMA Network Open*.

Supplemental Results 1. Propensity-score matching in the study cohort

**Cohort 1 and cohort 2 patient count before and after propensity score matching**		
Cohort	Patient count before matching	Patient count after matching
1 – GLP-1 RA users	473	460
2 – GLP1-RA non-users	4,398	460

Propensity score density function - Before and after matching (cohort 1 - purple, cohort 2 - green)

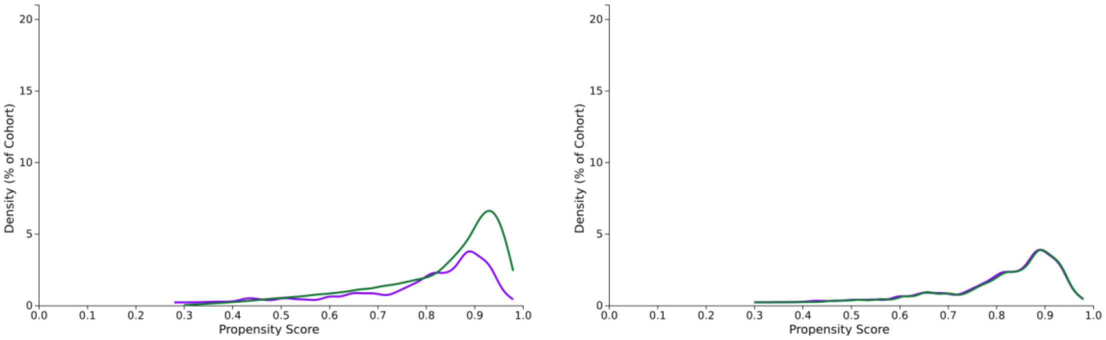

© 2025 Chi KY et al. *JAMA Network Open*.

Table S1. Association between GLP-1 RA and other CV endpoints in the matched cohort

Cardiac arrhythmia, %	21.7	27.0	0.77 (0.59 to 1.00)	0.053
Conduction disorder, %	10.4	11.7	0.86 (0.58 to 1.27)	0.46
Arterial/venous thromboembolism, %	7.4	10.7	0.68 (0.44 to 1.06)	0.090
Aortic or valvular heart disease, %	12.6	17.8	0.67 (0.48 to 0.94)	0.023
Cor pulmonale, %	7.0	10.7	0.63 (0.40 to 0.99)	0.043
Peripheral artery disease, %	11.7	13.0	0.88 (0.61 to 1.28)	0.52

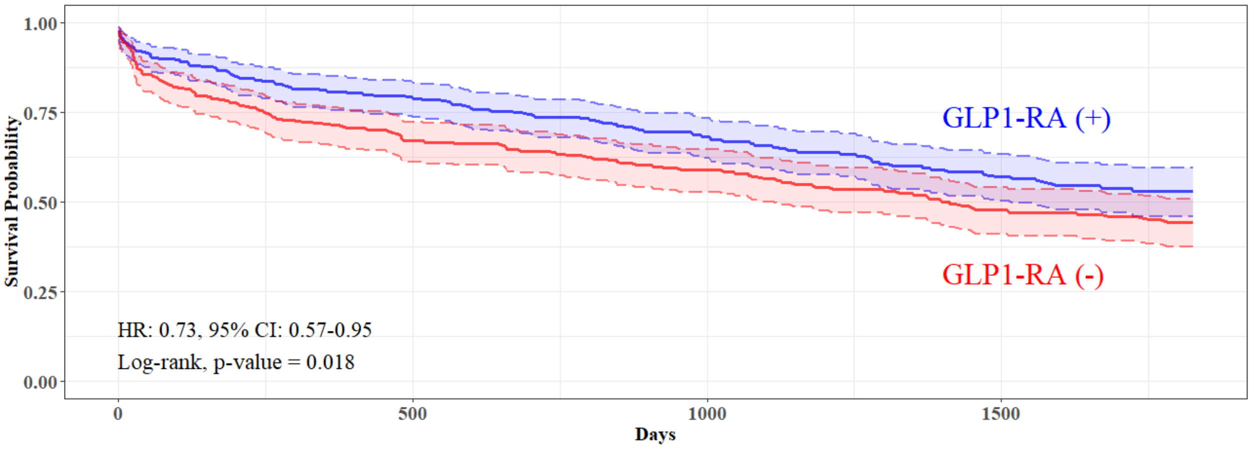

Figure S1. Kaplan-Meier survival curves for comparative major cardiac and cerebrovascular events in MGUS patients with type 2 diabetes treated with or without baseline GLP-1 RA.

© 2025 Chi KY et al. *JAMA Network Open*.

Table S2. Sensitivity analysis: cox proportional hazard analysis for the association between GLP-1 RA and study endpoints in the cohort excluding patients with DPP-4i and SGLT2i

	GLP-1 RA		HR (95% CI)	P-value (log-rank)
	Users (n = 288)	Non-users (n = 288)		
Primary endpoint				
MACCE, No (%)	111 (39.3)	134 (47.5)	0.73 (0.57 to 0.95)	0.01
Secondary endpoints				
All-cause mortality, No (%)	27 (9.5)	52 (18.4)	0.50 (0.31 to 0.79)	0.002
New-onset HF, No (%)	69 (24.4)	95 (33.6)	0.66 (0.48 to 0.95)	0.009
Decompensated HF, No (%)	36 (12.7)	57 (20.2)	0.58 (0.38 to 0.88)	0.009
Incident stroke/TIA, No (%)	27 (9.5)	35 (12.4)	0.72 (0.43 to 1.18)	0.19
Incident ACS, No (%)	26 (9.2)	21 (7.4)	1.22 (0.69 to 2.17)	0.49
AKI or Progression to ESRD, No (%)	88 (31.2)	113 (40.0)	0.71 (0.53 to 0.94)	0.01
Other CV events, No (%)	130 (45.1)	148 (51.3)	0.78 (0.61 to 0.98)	0.03

© 2025 Chi KY et al. *JAMA Network Open*.

© 2025 Chi KY et al. *JAMA Network Open*.

© 2025 Chi KY et al. *JAMA Network Open*.
