# Supplementary material for: GLP-1 RA Use and Major Adverse Cardiovascular Events in Patients With Monoclonal Gammopathy of Undetermined Significance
Source: JAMA Netw Open. 2025 Jun 30;8(6):e2517541. doi: 10.1001/jamanetworkopen.2025.17541 (PMC12210086; doi:10.1001/jamanetworkopen.2025.17541)
Supplement: Supplement 2. — Data Sharing Statement [file jamanetwopen-e2517541-s002.pdf]

# Data Sharing Statement

Chi. GLP-1 RA Use and Major Adverse Cardiovascular Events in Patients With Monoclonal Gammopathy of Undetermined Significance. *JAMA Netw Open*. Published June 30, 2025. doi:10.1001/jamanetworkopen.2025.17541

**Data**
**Data available:** No
